# Dysfunctional accessory gene regulator (*agr*) as a prognostic factor in invasive *Staphylococcus aureus* infection: a systematic review and meta-analysis

**DOI:** 10.1038/s41598-020-77729-0

**Published:** 2020-11-26

**Authors:** Soon Ok Lee, Shinwon Lee, Jeong Eun Lee, Kyoung-Ho Song, Chang Kyung Kang, Yu Mi Wi, Rafael San-Juan, Luis E. López-Cortés, Alicia Lacoma, Cristina Prat, Hee-Chang Jang, Eu Suk Kim, Hong Bin Kim, Sun Hee Lee

**Affiliations:** 1 Department of Internal Medicine, Pusan National University School of Medicine and Medical Research Institute, Pusan National University Hospital, 179 Gudeok-ro, Seo-gu, Busan, 49241 Republic of Korea; 2 Department of Internal Medicine, Seoul National University College of Medicine and Seoul National University Bundang Hospital, Seongnam, Republic of Korea; 3 Department of Internal Medicine, Seoul National University College of Medicine and Seoul National University Hospital, Seoul, Republic of Korea; 4 Division of Infectious Diseases, Samsung Changwon Hospital, Sungkyunkwan University School of Medicine, Changwon, Republic of Korea; 5 Unit of Infectious Diseases, University Hospital 12 de Octubre, Instituto de Investigación Hospital “12 de Octubre” (i+12), Universidad Complutense, Avenida de Córdoba, s/n, Madrid, Spain; 6 Unidad Clínica de Enfermedades Infecciosas, Microbiología y Medicina Preventiva, Hospital Universitario Virgen Macarena/Departamento de Medicina, Universidad de Sevilla/Instituto de Biomedicina de Sevilla, Sevilla, Spain; 7 Microbiology Department, Hospital Universitari Germans Trias i Pujol, Institut d’ Investigació Germans Trias i Pujol, CIBER Enfermedades Respiratorias (CIBERES), Universitat Autònoma de Barcelona, Badalona, Spain; 8 Julius Center for Health Sciences and Primary Care, University Medical Center Utrecht, Utrecht University, Utrecht, The Netherlands; 9 Department of Infectious Diseases, Chonnam National University Medical School, Gwang-ju, Republic of Korea

**Keywords:** Infectious diseases, Bacterial infection

## Abstract

The accessory gene regulator (*agr*) locus of *Staphylococcus aureus* is a quorum-sensing virulence regulator. Although there are many studies concerning the effect of dysfunctional *agr* on the outcomes of *S. aureus* infection, there is no systematic review to date. We systematically searched for clinical studies reporting outcomes of invasive *S. aureus* infections and the proportion of dysfunctional *agr* among their causative strains, and we performed a meta-analysis to obtain estimates of the odds of outcomes of invasive *S. aureus* infection with dysfunctional versus functional *agr*. Of 289 articles identified by our research strategy, 20 studies were meta-analysed for crude analysis of the impact of dysfunctional *agr* on outcomes of invasive *S. aureus* infection. Dysfunctional *agr* was generally associated with unfavourable outcomes (OR 1.32, 95% CI 1.05–1.66), and the impact of dysfunctional *agr* on outcome was more prominent in invasive methicillin-resistant *S. aureus* (MRSA) infections (OR 1.54, CI 1.20–1.97). Nine studies were meta-analysed for the impact of dysfunctional *agr* on the 30-day mortality of invasive *S. aureus* infection. Invasive MRSA infection with dysfunctional *agr* exhibited higher 30-day mortality (OR 1.40, CI 1.03–1.90) than that with functional *agr*. On the other hand, invasive MSSA infection with dysfunctional *agr* exhibited lower 30-day mortality (OR 0.51, CI 0.27–0.95). In the post hoc subgroup analysis by the site of MRSA infection, dysfunctional *agr* was associated with higher 30-day mortality in MRSA pneumonia (OR 2.48, CI 1.17–5.25). The effect of dysfunctional *agr* on the outcome of invasive *S. aureus* infection may vary depending on various conditions, such as oxacillin susceptibility and the site of infection. Dysfunctional *agr* was generally associated with unfavourable clinical outcomes and its effect was prominent in MRSA and pneumonia. Dysfunctional *agr* may be applicable for outcome prediction in cases of invasive MRSA infection with hardly eradicable foci such as pneumonia.

## Introduction

*Staphylococcus aureus* is a major pathogen responsible for invasive infections such as bacteraemia, endocarditis, osteomyelitis, arthritis, and pneumonia. Invasive *S. aureus* infection is still associated with high mortality and morbidity, and recent studies have shown that the mortality rates of *S. aureus* bacteraemia (SAB) are 20–30%, even though antibiotic therapies are advanced^1–5^. Many studies have shown that the persistent infection despite the use of susceptible antibiotics is associated with poor clinical outcomes^6–11^. To improve the outcomes of invasive *S. aureus* infection, it is essential to understand how *S. aureus* establishes and maintains infection in the host for a long time.

*Staphylococcus aureus* utilizes virulence factors to establish and maintain infection, depending on its growth phase^12–17^. Among the various virulence factors of *S. aureus*, the accessory gene regulator (*agr)* locus, which is a quorum-sensing virulence regulator, can play an important role in perpetuating infection^16,17^. At high cell density, the *agr* quorum-sensing circuit leads to decreased production of cell-wall-associated factors, causing the dispersion of the biofilm, the spread of the infection and a simultaneous increase in exoproteins, including protease, haemolysin, and super-antigen production^18^. The *agr* locus also leads to increased production of many murein hydrolases that are involved in autolysis^19^. Therefore, the dysfunction of the *agr* locus can cause the strain to form abundant biofilms and become deficient in autolysis even though the bacterial density is high. These changes can contribute to the persistence of the infection by hindering the host immune system.

According to recent studies, the alteration of *agr* function can result in decreased activity of various antibiotic agents against *S. aureus*. The attenuation of the bactericidal activity of vancomycin against *S. aureus*, the increased minimum inhibitory concentration of vancomycin and the development of vancomycin intermediate/resistant *S. aureus* (VISA) phenotypes are associated with dysfunctional *agr*^7,20–26^. Dysfunctional *agr* of *S. aureus* can affect the inoculum effect of methicillin-sensitive *S. aureus* (MSSA) against beta-lactam antibiotics and reduce susceptibility to daptomycin^27,28^.

Many clinical studies have also reported the influence of dysfunctional *agr* on the courses of invasive *S. aureus* infection, and some studies have shown that dysfunctional *agr* may be related to unfavourable outcomes such as persistent bacteraemia and a high mortality rate^7,29–32^. The information, however, is conflicting and can be affected by various conditions and situations in which the studies were performed. However, no study that systematically reviews and quantitatively analyses the studies concerning the association between *agr* dysfunction and clinical outcomes has been performed so far.

The aim of this study was to perform a systematic literature review and meta-analysis to measure the association between the dysfunction of the *agr* locus and clinical outcomes in patients with invasive *S. aureus* infection. We performed subgroup analysis evaluating MRSA and MSSA as well as different sites of infection.

## Results

**Figure 1:**
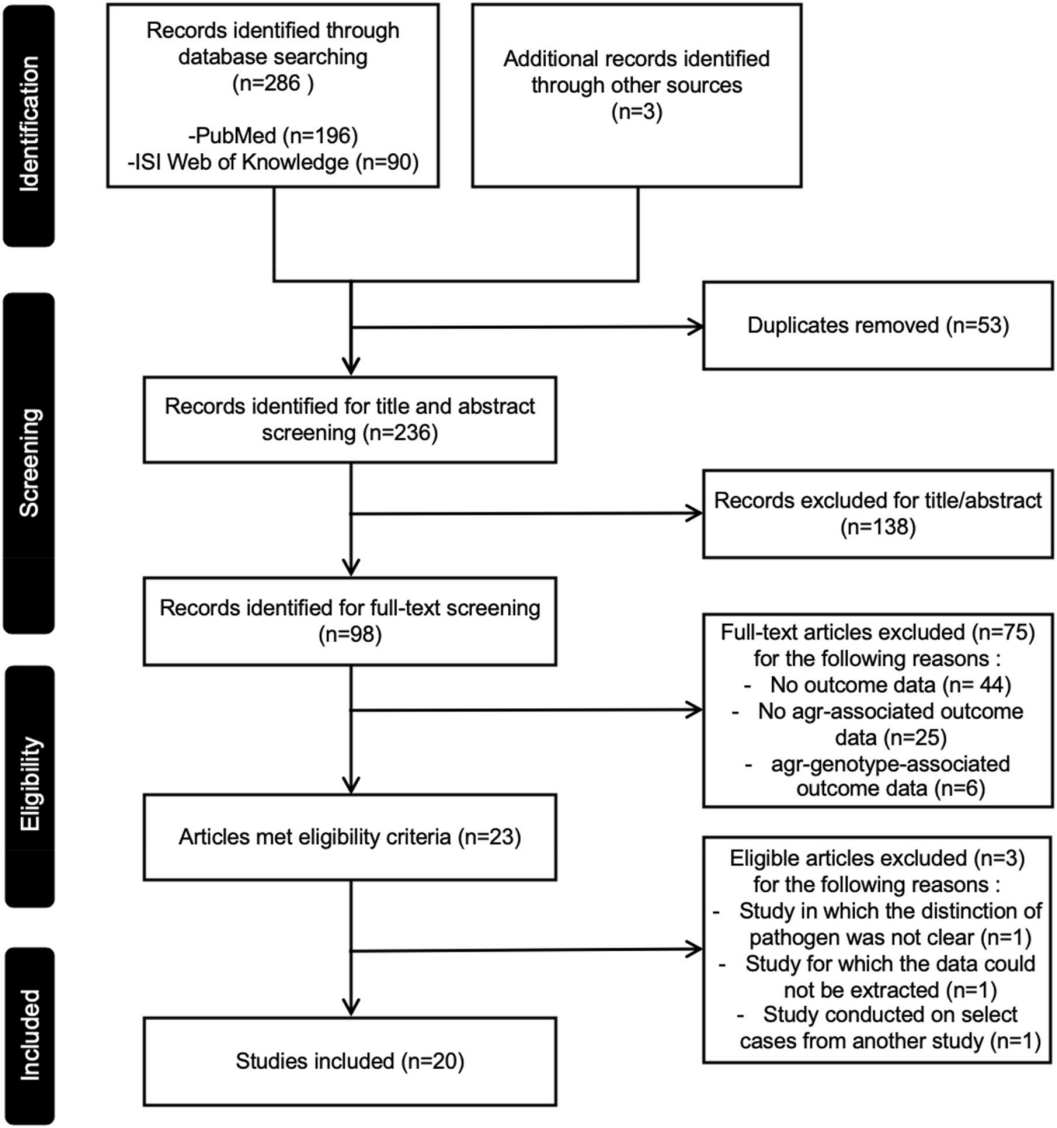
PRISMA flow diagram of the study identification and selection process for outcome analysis (modified from Moher et al.)^33^.

The database search identified 286 articles, and we identified three more articles. Afterwards, 53 studies were removed due to duplication and 215 studies were excluded due to a lack of relevant information regarding our redefined outcome parameters and insufficient design. Finally, 20 studies were included in our analyses (Fig. 1). Among the 20 included studies, 18 studies collected cases of bacteraemia and their blood isolates, and two studies collected cases of lower respiratory infections and their respiratory isolates. Among 18 bacteraemia studies, seven studies used the isolates that were consecutively collected, and seven studies selected the cases and isolates by specific conditions other than the site of infection; infection with persistent bacteraemia, infection by MRSA with vancomycin MIC = 2 mg/L, or ICU setting. Four studies selected cases and isolates by specific sites of infection: catheter-related infection, infection with removed eradicable foci and without metastatic infection, endocarditis, or pneumonia.

Nineteen studies identified dysfunctional *agr* isolates by measuring delta-hemolysin production according to the method of Sakoulas G et al.^21^, and 1 study identified dysfunctional *agr* isolates by *agr* CAMP assay and the vesicle lysis test^34^.

Four studies included all cases of invasive *S. aureus* infections regardless of oxacillin susceptibility, 12 studies included only MRSA cases, and four studies included only MSSA. Twelve studies reported mortality as their main outcome, five studies reported the persistent bacteraemia as their main outcome, and 12 studies reported treatment failure (composite outcomes). Detailed characteristics of the included studies are provided in Table 1.

**Table 1:** Studies in which the association between *agr* dysfunction and treatment outcomes of invasive *Staphylococcus aureus* infections was able to be evaluated.

Study and publication year	Location	Study period	Isolate No., MR/MS (%)	No. of centres	Inclusion/study setting	No. (%) of IE	Main therapeutic agents (%)	Proportion of agr dysfunction according to outcomes (no. of agr dysfunction/total no.)	Proportion of high VM MIC (%)
**Studies that collected cases and isolates consecutively**									
Schweizer (2011)^29^	USA	2003–2007	814, MR(60)/MS(40)	1	SAB, adult/retrospective	138 (17)	VM (86)	Death (30 day) 33/109 vs. Survival 149/705	MIC ≥ 1.5 (76.2)
Chong (2013)^35^	Korea	2008–2010	159, MR (100)	1	MRSAB excluding intermediate duration, Adult/Prospective	NR	VM (92.8), TP (3.6), LZ (3.6)	PB 44/65 vs. NPB 63/94^d^	NR
Jang (2013)^24^	Korea	2005–2008	307, MR (100)	2	MRSAB (≥ 16 years)/retrospective	2 (0.7)	GP (75.2)	Death (30 day) 36/98 vs. Survival 72/209	MIC = 2 (12.7), hVISA (6.2)
Wi (2015)^27^	Korea	2011–2012	146, MS (100)	9	MSSAB/prospective	19 (13)	^f^BLT (69.2), BLT + GP (21.9), GP (2.1)	Death (30 day) 2/33 vs. Survival 17/113	NR
Kang (2015)^31^	Korea	2009–2013	171, MR (100)	1	MRSAB (≥ 15 years)/prospective	NR	^f^VM (99.4), LZ (0.6)	Death (SAB-attributable) 34/44 vs. Survival 72/127^d^	NR
López-Cortés (2015)^36^	Spain	2008–2011	135, MS (100)	1	MSSAB (≥ 18 years)/prospective	NR	^f^BTL (87.6), GP (6.2), others (6.2)	1) Death (30 day) 23/37 vs. Survival 47/98 2) PB 14/26 vs. NPB 48/99	MIC ≥ 1.5 (21.5)
Sullivan (2017)^37^	USA	2010–2012	252, MS (100)	1	MSSAB (≥ 18 years)/Retrospective	NR	^g^VM (66.5), BLT (27.3), others (6.2)	Death (30 day) 2/45 vs. Survival 18/207	MIC ≥ 2 (33.3)
**Studies that collected cases and isolates by specific conditions other than the site of infection**									
Fowler (2004)^7^	USA	1995–2000	39, MR (100)	1	SAB (≥ 18 years), All PB (n = 21) and randomly selected NPB (n = 18)	9 (23.1)	VM (97.4), [adjunctive AG (33.3), RF (10.3)]	PB 15/21 vs. NPB 7/18	NR
Moise (2007)^38^^,a^	USA	1998–2002	34, MR (100)	6	Randomly selected agr II MRSAB and matched non-agr-II MRSAB	0 (0)	VM^b^	PB 14/16 vs. NPB 11/18	NR
McCalla (2008)^39^^,b^	USA	2002–2005	89, MR (100)	M	MRSAB from clinical trial comparing DM (n = 45) vs. standard treatment (n = 44)/Post hoc analysis^40^	NR	DM (50.6), VM + AG (49.4)	Failure 16/55 vs. Cure 9/34	NR
Walraven (2011)^41^	USA	2002–2009	139, MR (100)	1	MRSAB (≥ 18 years) and received VM/retrospective	29 (20.9)	VM (100)	Failure 13/67 vs. Cure 13/72	MIC ≥ 1.5 (92.1)
Casapao (2013)^42^	USA	2004–2012	122, MR (100)	5	hVISAB (n = 61) and matched VSSAB (n = 61)/retrospective	48 (39.3)	VM (100)	Failure 15/70 vs. Success 7/52	hVISA (50)
Hu (2015)^43^	Taiwan	2009–2010	48, MR (100)	1	MRSAB & treated in ICUs (≥ 18 years)/retrospective	NR	NR	Death (in hospital) 12/35 vs. Survival 1/13	hVISA (27.1)
Kang (2017)^32^	Korea	2009–2016	152, MR (100)	11	Persistent SAB among 960 MRSAB (≥ 15 years)/prospective	11 (7.2)	^g^VM (90.1), [adjunctive RF (10.5)]	Death (in hospital) 34/50 vs. Survival 50/102^d^	MIC ≥ 1.5 (56.6), hVISA (7.2)
Yang (2018)^44^	Taiwan	2009–2012	147, MR (100)	1	High VM MIC(= 2 mg/L) MRSAB/Retrospective	NR	DM (37.4), GM (54.4), Others (8.2)	Failure 24/79 vs. Success 17/68 Death (30 day) 16/47 vs. Survival 25/100	hVISA (37.4)
**Studies that collected cases and isolates by the specific site of infection**									
Sharma-Kuinkel (2012)^45^	USA	2005–2007	287, MR (60)/MS (40)	M	*S. aureus* LRTI from clinical trial comparing TV vs. VM/Post hoc analysis^46^	NR	VM, TV	Failure 19/34^47^, 3/18^9^ vs. Cure 60/138^47^, 13/96^9^	NR
Park (2013)^30^,^e^	Korea	2008–2010	87, MR (100)	1	SAB with the removal of eradicable foci and without metastatic infections, Adult/Prospective/Post hoc analysis^35^	NR	VM (95.4), LZ or TP (4.6)	PB 29/31 vs. NPB 42/56	MIC ≥ 1.5 (48.3)
McDanel (2015)^48^	USA	2003–2010	75, MR (100)	2	MRSA LRTI and treated with initial VM or LZ/Retrospective	NR	VM (81.4), LZ (5.3), VM + LZ (13.3)	Death (30 day) 4/18 vs. survival 9/57	NR
Gomes-Fernandes (2017)^34^	Spain	NR	18, MR (5.6)/MS(94.4)	1	*S. aureus* LRTI and control groups (nasal carriage and bacteraemia^c^)/Retrospective	NR	NR	(1) PB 1/3 vs NPB 2/15 (2) Death 1/8 vs. Survival 2/10^d^	
San-Juan (2017)^49^	Spain	2011–2014	83, MS (100)	5	*Central-line-associated* MSSAB/prospective	NR	NR	Complication 5/24 vs. No complication 13/56	NR
Fernández-Hidalgo (2018)^50^	Spain	2013–2016	213, MS (81)/MR (19)	15	*S. aureus* IE (≥ 18 years)/Prospective	213 (100)	NR	Death (in hospital) 12/58^9^, 7/21^47^ vs. Survival 28/115^9^, 9/19^47^	MIC ≥ 1.5 (35.7)

*MRSA (B)*, methicillin-resistant *Staphylococcus aureus* (bacteraemia); *MSSA (B)*, methicillin-susceptible *Staphylococcus aureus* (bacteraemia); *hVISA (B)*, heterogeneous vancomycin intermediate *Staphylococcus aureus* (bacteraemia); *VSSA (B)*, vancomycin susceptible *Staphylococcus aureus* (bacteraemia); *SAB*, *Staphylococcus aureus* bacteraemia; *PB*, persistent bacteraemia; *NPB*, non-persistent bacteraemia; *IE*, infective endocarditis; *LRT (I)*, lower respiratory tract (infection); *NR*, not reported; *CNS*, central nervous system; *MIC*, minimal inhibitory concentration; *IQR*, interquartile range; *ICU*, intensive care unit; *GP*, glycopeptide; *VM*, vancomycin; *TP*, teicoplanin; *TV*, telavancin; *AG*, aminoglycoside; *RF*, rifampin; *DM*, daptomycin; *LZ*, linezolid; *BLT*, beta-lactam.

^a^*agr* functionality was measured by *agr* score in this study, *agr* score 0–1 was considered as *agr* dysfunction and *agr* score 2–4 as *agr* function.

^b^There is a record of the vancomycin trough level of each group, but there is no record of definite antibiotic use.

^c^Excluded LRTI cases in our analysis because colonization cases were mixed.

^d^Analysed by 30-day mortality as an outcome using information from the researchers of the primary studies.

^e^Excluded from analysis because this study was conducted on selected cases from the study of Chong et al.

^f^Studies reported definitive therapy.

^g^Initial therapy as their main therapeutic agents; otherwise, therapies were not reported.

### Overall unfavourable outcomes

**Figure 2:**
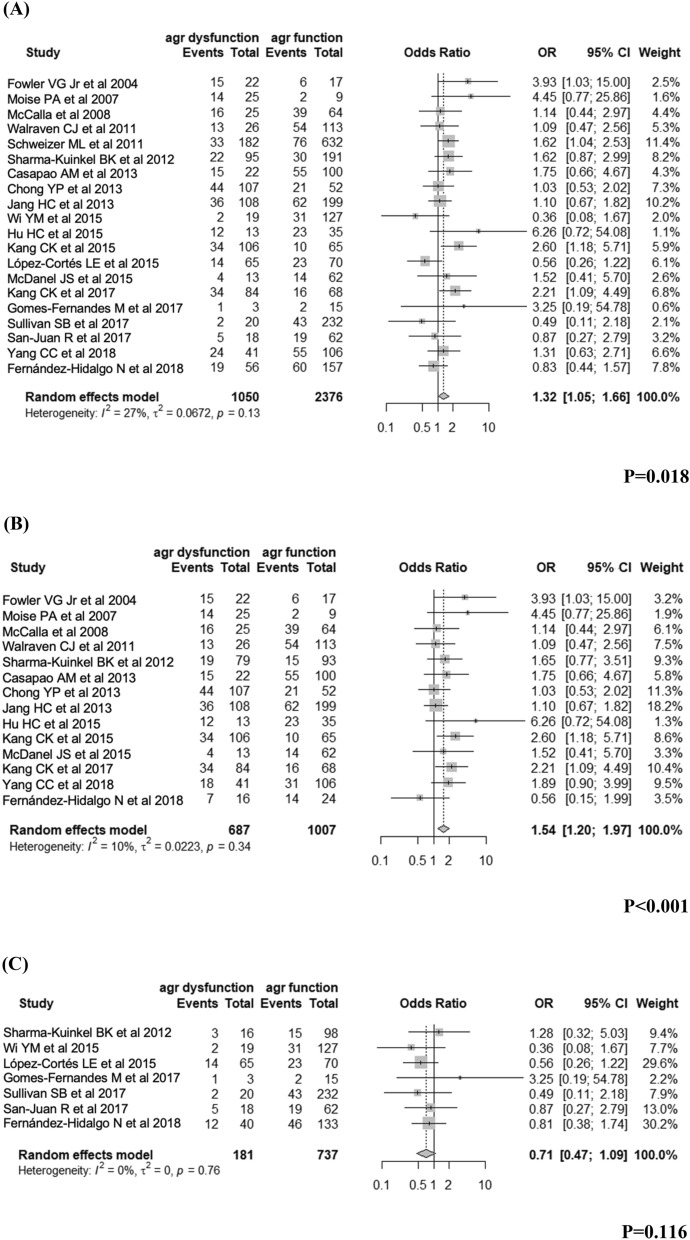
The results for the association of *agr* dysfunction with overall outcome in patients with invasive *S. aureus* infection: (**A**) total, (**B**) MRSA, and (**C**) MSSA.

For the association between dysfunctional *agr* and overall unfavourable outcomes of invasive *S. aureus* infection, data were available for 20 studies comprising 3426 patients (Fig. 2). To estimate the crude overall tendency, we conducted a meta-analysis using all included studies, although treatment failure was variably defined in each study^7,30,38,39,41,42,44,45,49^.

Pooled analysis showed that invasive *S. aureus* infection with dysfunctional *agr* was significantly associated with unfavourable outcomes (OR 1.32, 95% CI 1.05–1.66, *I*^*2*^ = 0.27, Fig. 2A). The analysis of fourteen studies with MRSA demonstrated that invasive MRSA infection with dysfunctional *agr* exhibited an increased likelihood of unfavourable outcomes, and the effect was statistically significant (OR 1.54, 95% CI 1.20–1.97, *I*^2^ = 0.10, Fig. 2B), while the analysis of seven MSSA studies demonstrated that invasive MSSA infection with dysfunctional *agr* did not increase the likelihood of unfavourable outcomes nor was it statistically significant (OR 0.71, 95% CI 0.47–1.09, *I*^2^ = 0, Fig. 2C).

### Mortality

For the association between dysfunctional *agr* and the mortality of invasive *S. aureus* infection, data were available for 13 studies comprising 2659 patients, and pooled analysis showed that invasive *S. aureus* infection with dysfunctional *agr* significantly increased the likelihood of death in MRSA but decreased the likelihood of death in MSSA (Supplementary Fig. 1).

**Figure 3:**
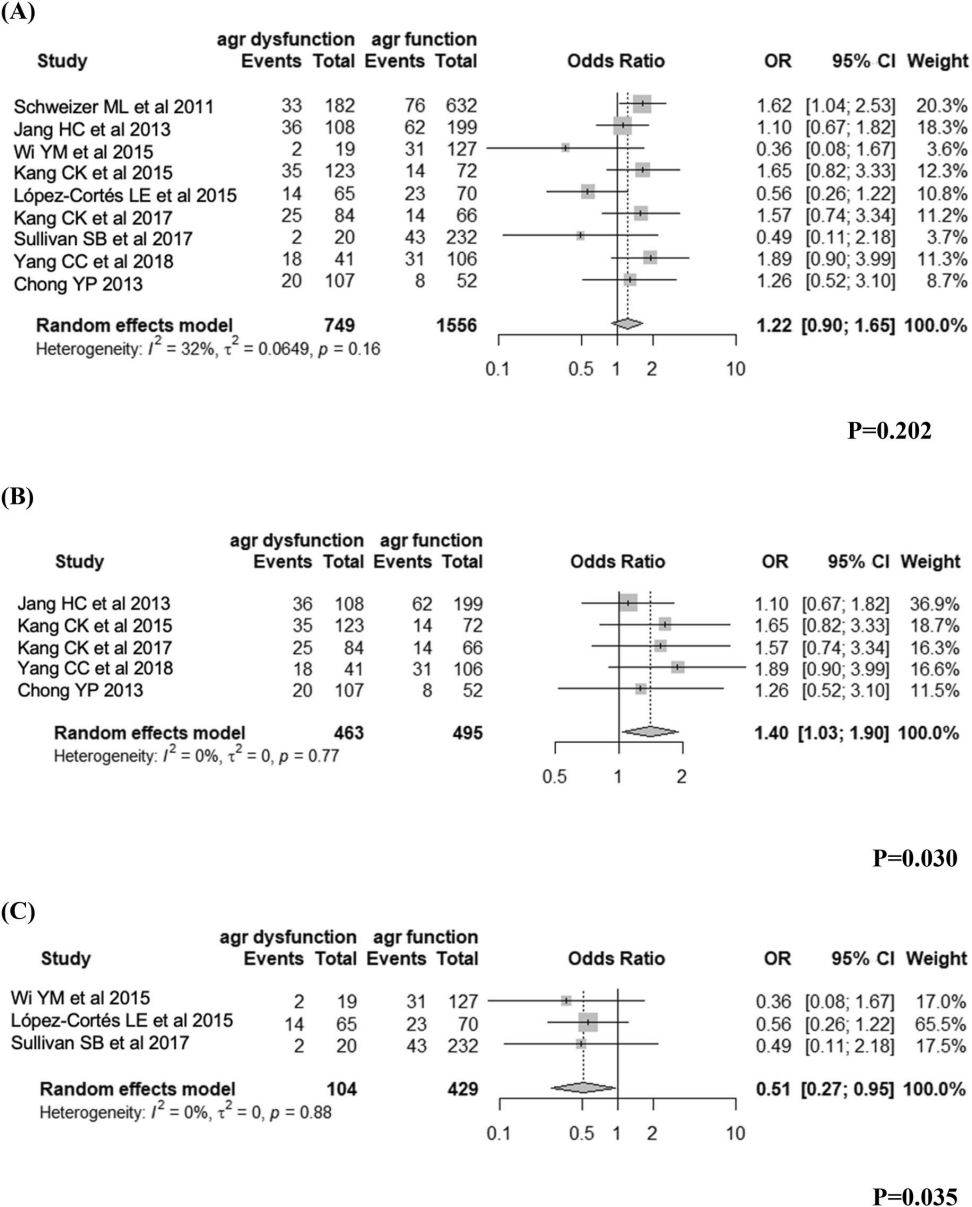
The results for the association of *agr* dysfunction with 30-day mortality in patients with invasive *S. aureus* infection: (**A**) total, (**B**) MRSA, and (**C**) MSSA.

For the association between dysfunctional *agr* and 30-day mortality of invasive *S. aureus* infection, we included studies that reported or provided 30-day mortality as an outcome and that did not selectively include patients with specific sites of infection; data were available for nine studies comprising 2305 patients (Fig. 3). Pooled analysis showed that invasive *S. aureus* infection with dysfunctional *agr* was not associated with higher 30-day mortality in analyses that did not consider oxacillin susceptibility (OR 1.22, 95% CI 0.90–1.65, *I*^2^ = 0.32, Fig. 3A). However, in the subgroup analysis of five studies with MRSA, invasive MRSA infection with dysfunctional *agr* showed significantly higher 30-day mortality than that with functional *agr* (OR 1.40, 95% CI 1.03–1.90, *I*^2^ = 0, Fig. 3B). On the other hand, invasive MSSA infection with dysfunctional *agr* showed lower 30-day mortality than that with functional *agr* (0.51, 95% CI 0.27–0.95, *I*^2^ = 0, Fig. 3C).

### Mortality by the site of infection

We investigated the association between dysfunctional *agr* and 30-day mortality of invasive *S. aureus* infection by sites of infections for the five most common infection sites: central-line-associated bloodstream infection (CLABSI), pneumonia, skin and soft tissue infection (SSTI), bone and joint infection (BJI) and endocarditis (Supplementary Table 2).

**Figure 4:**
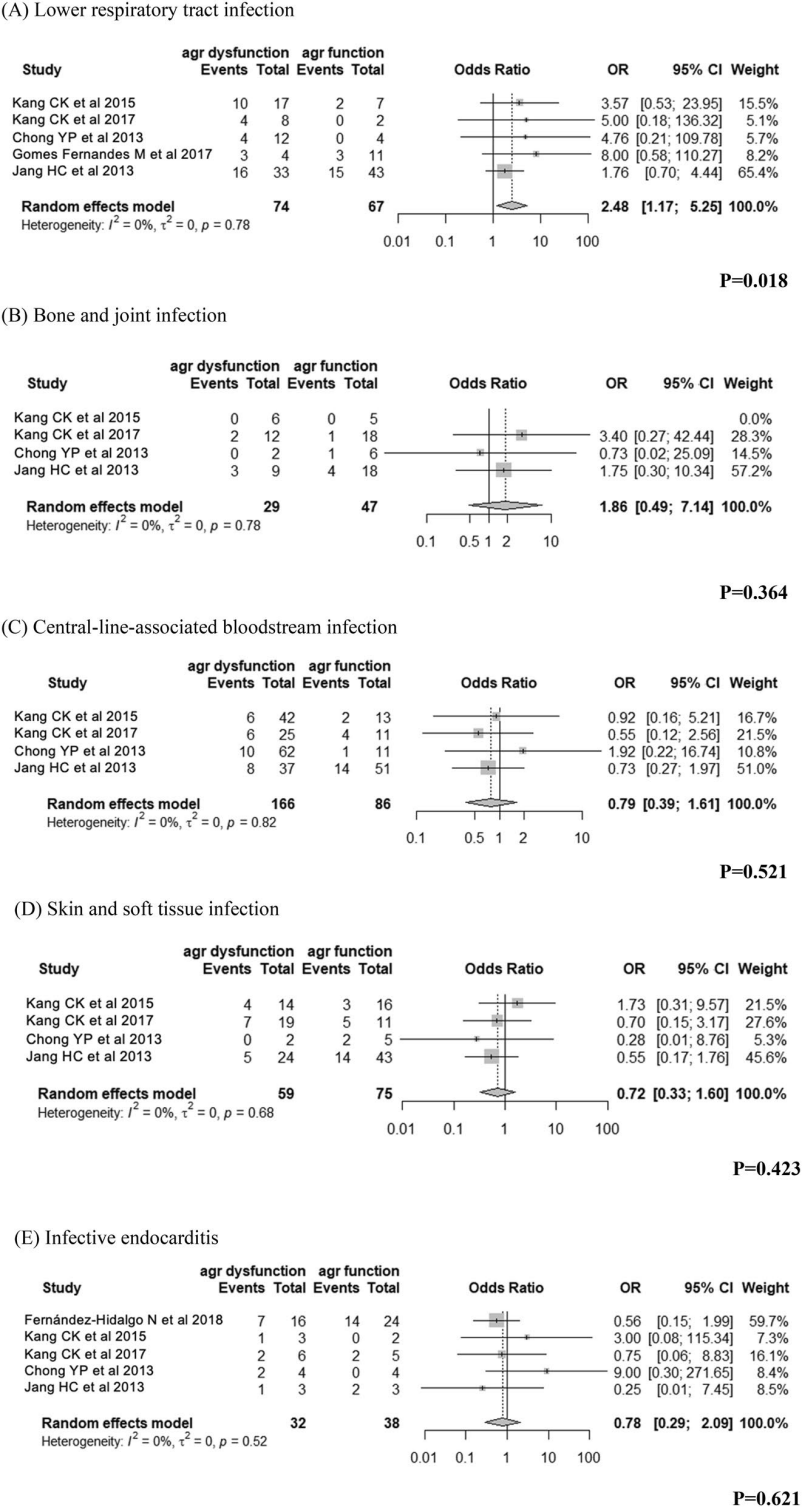
The association of *agr* dysfunction with mortality in patients with invasive MRSA infection according to the site of infection.

Pooled analysis showed that dysfunctional *agr* was associated with higher 30-day mortality than functional *agr* in MRSA pneumonia (OR 2.48, 95% CI 1.17–5.25, *I*^*2*^ = 0, Fig. 4A). BJI with MRSA with dysfunctional *agr* (OR 1.86, 95% CI 0.49–7.14, Fig. 4B) also had an increased likelihood of death within 30 days, while CLABSI with MRSA with dysfunctional *agr* (OR 0.79, 95% CI 0.39–1.61, Fig. 4C) and SSTI with MRSA with dysfunctional *agr* (OR 0.72, 95% CI 0.33–1.60, Fig. 4D) had a decreased likelihood of death within 30 days, and both results were not statistically significant. And infective endocarditis with MRSA with dysfunctional *agr* (OR 0.78, 95% CI 0.29–2.09, Fig. 4E) also had an decreased likelihood of death within 30 days, and the result was not statistically significant.

Pooled analysis showed that CLABSI with MSSA with dysfunctional *agr* had a decreased likelihood of death within 30 days, and BJI with MSSA with dysfunctional *agr* had an increased likelihood of death within 30 days, and both results were not statistically significant (Supplementary Fig. 2).

### Persistent bacteraemia

**Figure 5:**
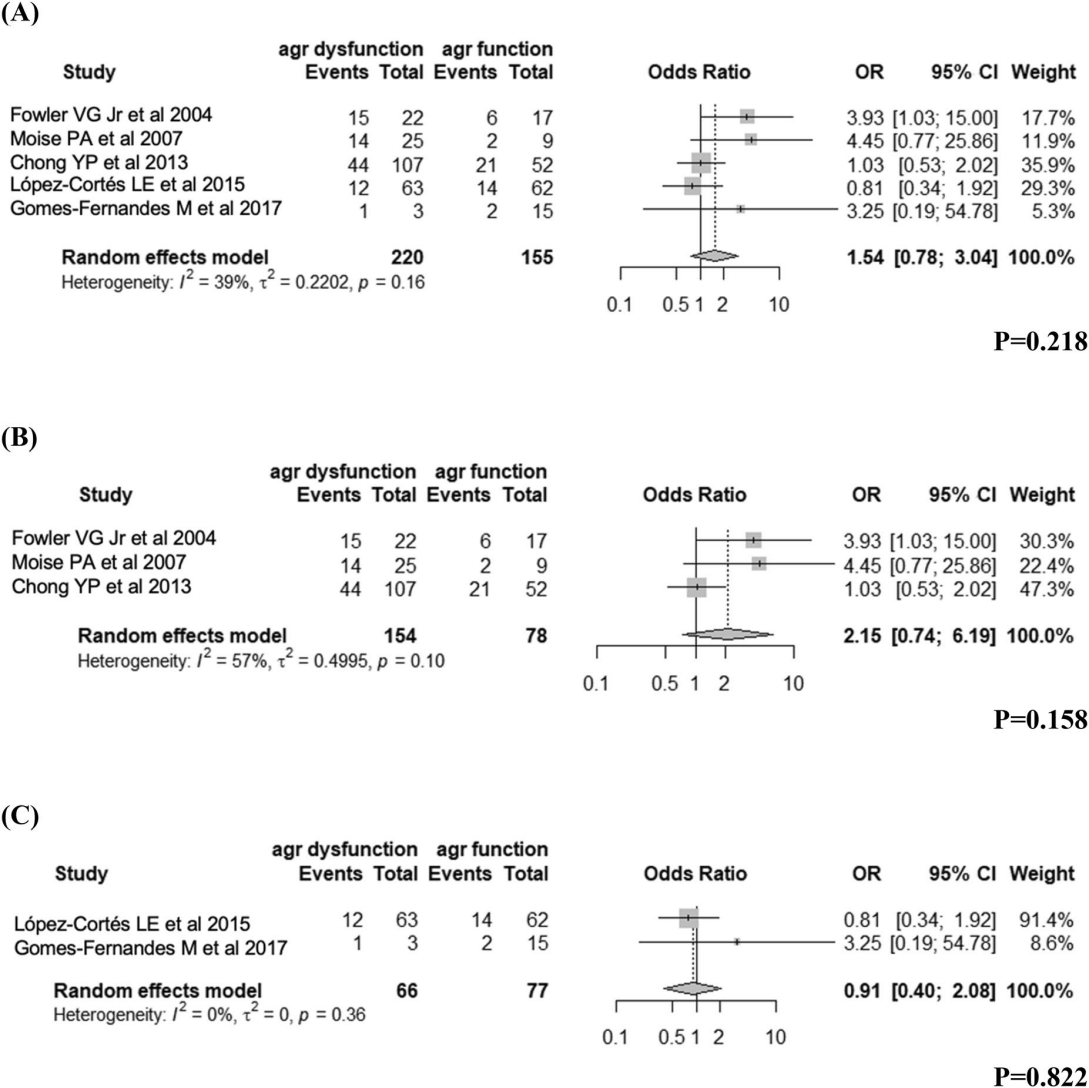
The results for the association of *agr* dysfunction with persistent bacteraemia in patients with *S. aureus* bacteraemia: (**A**) total, (**B**) MRSA, and (**C**) MSSA.

Among the 18 SAB studies, five studies considered the persistent bacteraemia as an outcome, comprising 375 patients. Pooled analysis showed that SAB with dysfunctional *agr* was generally not associated with persistent bacteraemia (OR 1.54, 95% CI 0.78–3.04, *I*^*2*^ = 0.39, Fig. 5A). In the subgroup analysis of three studies of MRSA SAB, we observed an increased likelihood of persistent bacteraemia, but the increase was not statistically significant (OR 2.15, 95% CI 0.74–6.19, *I*^2^ = 0.57, Fig. 5B). In the subgroup analysis of 2 studies of MSSAB, we observed no difference in the rates of persistent bacteraemia between the dysfunctional *agr* group and the functional *agr* group (OR 0.91, 95% CI 0.40–2.08, *I*^2^ = 0, Fig. 5C).

## Discussion

To date, many studies have analysed the association between *agr* dysfunction and poor clinical outcomes of invasive *S. aureus* infection in various clinical settings^30,32,43,44,49,50^. Since Schweizer et al. reported that SAB with dysfunctional *agr* was associated with excessive mortality among severely ill patients^29^, subsequent studies have demonstrated that *agr* dysfunction was associated with higher mortality^31,32^, and the persistence of bacteraemia in patients with SAB, especially in MRSA bacteraemia^7,30^. However, some studies reported that *agr* dysfunction of MRSA bacteraemia was not associated with treatment failure^39,41,42^, or mortality^51^.

Despite the fact that various studies have addressed the association between *agr* dysfunction and the outcome of invasive *S. aureus* infection, data on its role in different infections and populations remained scattered. Despite the necessity for the integration of the information, this subject has never been systematically reviewed. Therefore, we systematically reviewed and performed a meta-analysis of 20 studies focusing on the association between clinical outcomes and dysfunctional *agr* in invasive *S. aureus* infections to investigate whether *agr* dysfunction can be a marker of poor clinical outcome. We performed data analysis using three different outcomes, namely overall unfavourable outcomes, 30-day mortality and persistent bacteraemia. To reduce the complexity due to variably defined outcomes, we performed subgroup analysis by the site of infection using 30-day mortality.

This meta-analysis demonstrated that invasive *S. aureus* infection with dysfunctional *agr* significantly increased the likelihood of unfavourable outcomes. However, the implication of *agr* dysfunction on the outcomes of invasive *S. aureus* infections was not prominent. The reason for the modest implication of dysfunctional *agr* on outcomes of invasive *S. aureus* infection could be that the studies dealt with various end points such as mortality, treatment failure (composite outcomes) and the persistence of bacteraemia. Oxacillin susceptibility, one of most important confounding factors, can affect the outcomes of invasive *S. aureus* infection^52^. Therefore, we performed a meta-analysis with the studies that reported or provided information on 30-day mortality according to the functionality of the *agr* locus of causative strains to minimize the effect of variances of end points of studies. The analysis demonstrated that invasive *S. aureus* infection with dysfunctional *agr* showed an increased likelihood of 30-day mortality in MRSA and a decreased likelihood of 30-day mortality in MSSA. It was interesting that dysfunctional *agr* could affect outcomes of invasive *S. aureus* infection differently according to the oxacillin susceptibility of isolated strains.

The difference in the impacts of dysfunctional *agr* on MRSA and MSSA infections can be speculated as follows. First, various experimental and clinical studies suggested that *S. aureus* acquires the ability to maintain persistent infection due to dysfunctional *agr* but loses virulence at the cost of dysfunctional *agr*^25,52–56^. Second, the main therapeutic agents of studies with MSSA were anti-staphylococcal beta-lactam antibiotics. Antistaphylococcal beta-lactam antibiotics showed more rapid bactericidal effects than vancomycin in invasive *S. aureus* infection^57^. MSSA bacteraemia usually exhibits a shorter duration of persistence and less prevalently progresses to persistent bacteraemia^1,35^. The use of antistaphylococcal beta-lactam antibiotics may weaken the effect of persistent infection due to *agr* dysfunction. Therefore, it is possible that weakened virulence due to *agr* dysfunction nullifies the effect of persistent infections, and these effects may be more prominent in MSSA than in MRSA. A previous study demonstrated a relationship between isolates with reduced vancomycin susceptibility (RVS) and dysfunctional *agr* in MSSA bacteraemia^26^, and several studies demonstrated that infection caused by the RVS phenotype in MSSA isolates was associated with poor clinical outcomes regardless of antibiotics^37,58–61^. However, we observed that dysfunctional *agr* was significantly associated with lower mortality in invasive MSSA infections. Therefore, dysfunctional *agr* might not be a marker of poor clinical outcomes of invasive *S. aureus* infection when the causative organism is susceptible to oxacillin.

Depending on our findings, the 30-day mortality of invasive MRSA infection with dysfunctional *agr* was 1.4-fold higher than that of MRSA infection with functional *agr*. The implication of dysfunctional *agr* on the outcomes of invasive MRSA infections was statistically significant, but the magnitude of the effect of dysfunctional *agr* was not prominent. The lower prominence of the effect of dysfunctional *agr* on the outcomes of invasive MRSA infection in our meta-analysis can be explained by the fact that the included MRSA studies usually had other powerful confounding factors, such as a high proportion of community-acquired MRSA^38^, high vancomycin MIC^49^, hVISA phenotype^42^, different sites of infections (endocarditis or pneumonia)^41^, and the removal (or not) of foreign bodies^35^.

First, reduced vancomycin susceptibility (RVS) needs to be considered an important factor when we evaluate factors affecting unfavourable outcomes of invasive MRSA infections because intravenous administration of vancomycin is recommended as the standard treatment of patients with invasive MRSA infection; as a matter of fact, most of the included studies administered vancomycin as the main therapeutic agent for MRSA infections. A previous study reported that a high MIC of vancomycin is associated with high mortality and treatment failure in MRSA bacteraemia^62–64^, and *agr* dysfunction is associated with the attenuation of the bactericidal activity of vancomycin and the development of vancomycin intermediate/resistant *S. aureus* (VISA)^7,20,21,43^. This suggests that there is a possible intrinsic survival advantage of dysfunctional *agr* under vancomycin selective pressure^65–67^. However, most studies that used vancomycin as the main therapeutic agent showed a significant association between *agr* dysfunction and unfavourable outcomes regardless of vancomycin MIC^29–32^. Therefore, dysfunctional *agr* might be a marker of poor clinical outcomes of MRSA bacteraemia regardless of vancomycin MICs of causative organisms when patients were treated with vancomycin.

Second, the implication of *agr* dysfunction on unfavourable outcomes can be affected by specific conditions other than vancomycin MICs, such as the severity of infections and the proper removal of sources. Schweizer ML et al. demonstrated that *agr* dysfunction in SAB isolates was independently associated with high mortality among severely ill patients^29^. Hu HC et al. reported that *S. aureus* infection possessing dysfunctional *agr*, which was associated with the hVISA phenotype, exhibited markedly higher in-hospital mortality (12/13, 92.3%) than functional *agr* infection (23/35, 65.7%) in an ICU-setting study^43^. The study that limited the subjects to patients with adequate source control, which was another important factor for the outcome of invasive *S. aureus* infection, demonstrated that *agr* dysfunction is an independent risk factor for MRSA bacteraemia that persists despite the source control^30^. These findings suggested that dysfunctional *agr* can be a microbiological predictor of unfavourable outcomes in severe MRSA infection despite proper source control.

Third, the site of infection is one of most important factors in the outcomes of invasive *S. aureus* infection, and the implication of *agr* dysfunction on unfavourable outcomes can be affected by the site of infection. Therefore, we performed a subgroup analysis considering the site of infection. In the subgroup analysis by the site of infection, lower respiratory infection caused by MRSA with dysfunctional agr showed significantly higher mortality than MRSA with functional agr. BJI with MRSA with dysfunctional *agr* had an increased likelihood of mortality, although this result was not statistically significant. On the other hand, CLABSI and SSTI were not significantly affected by *agr* dysfunction. It is interesting that dysfunctional *agr* can affect the outcomes of invasive *S. aureus* infection differently according to the site of infection, although the reason for this difference is unclear. One probable explanation is as follows. First, patients with eradicable foci of infections, such as CLABSI and SSTI, might be more easily treated, and the magnitude of the effect of *agr* dysfunction on outcomes would be minimal. On the other hand, patients with foci that are difficult to eradicate, such as pneumonia and BJI, might be more difficult to treat, and the magnitude of the effect of dysfunctional foci would be more prominent^30^. Second, the vancomycin molecule is relatively large and penetrates poorly into the alveolar lining fluid and alveolar macrophages and into the bone and joints, further exacerbating the effect of RVS due to dysfunctional *agr*^68–70^.

The results of this meta-analysis should be interpreted with caution. First, the enrolled studies of our meta-analysis used different patient populations, different definitions of treatment failure, different definitions of the persistence of bacteraemia, and different time points of mortality. Except for seven studies that used consecutively collected isolates, the other enrolled studies used isolates from selected cases, such as specific sites of infection, persistent bacteraemia, a higher vancomycin MIC, and treatment in ICUs. The studies that used isolates from selected cases may have selected more virulent strains or those causing more difficult-to-treat infection, making it hard to compare the data with those of the studies that collected all consecutive isolates. To compensate for this heterogeneity of studies, we conducted subgroup analysis according to the sites of infection. We could extrapolate the effect of *agr* dysfunction on severity of disease based on the subgroup analysis by the site of infection. Moreover, the analysis was not adjusted for important factors that might affect clinical outcomes, such as source control, antibiotics used, time elapsed from infection onset to adequate therapy, incomplete vancomycin dosing, hVISA or strains with higher vancomycin MICs, severity of illness scoring, etc. Most of the included studies were retrospective, observational studies; therefore, publication bias is likely.

Second, most studies that investigated the association between *agr* dysfunction of MRSA bacteraemia and 30-day mortality were performed in Korea. In Korea, more than 70% of MRSA is SCC*mec* type II (mainly ST5), and 20–30% is SCCmec type IV/IVa (mainly ST72)^71^. Several molecular epidemiology studies reported that 41–90% of SCC*mec* type II-MRSA was associated with *agr* dysfunction, but 3–12% of SCCmec type IV/Iva-MRSA was associated with *agr* dysfunction in Korea^24,31,72,73^. Therefore, our results should be interpreted with caution because it is not clear whether the poor outcomes of invasive MRSA infection with dysfunctional *agr* in our results might be affected by the predominance of a specific clone, SCC*mec* type II (mainly ST5), which was associated with dysfunctional *agr*. Third, except for 1 study^34^, all included studies evaluated the activity of *agr* by the delta-haemolysin test. The interpretation of the delta-haemolysin test can be subject to low sensitivity^74^. However, Shopsin et al. performed a delta-haemolysin test and Northern blotting test for RNA III production and found that the delta-haemolysin test was a fair specific marker for dysfunctional *agr*^29,75^.

## Conclusion

Invasive *S. aureus* infection with dysfunctional *agr* was associated with unfavourable clinical outcomes. However, dysfunctional *agr* is not universally applicable to clinical decision making because dysfunctional *agr* can affect the clinical outcome of invasive *S. aureus* infection differently according to the oxacillin susceptibility profile. The implication of dysfunctional *agr* was also different according to the sites of infection. However, dysfunctional *agr* may be used as a predictor of outcomes of invasive *S. aureus* infection if the patients have pneumonia caused by MRSA. Further study is warranted to determine how dysfunctional *agr* affects oxacillin susceptibility differently and according to sites of infection in invasive *S. aureus* infection.

## Methods

### Search strategy and selection criteria

We searched for clinical studies reporting the proportion of dysfunctional *agr* and its association with outcomes of invasive *S. aureus* infections from database inception to 26th September 2018 in *Medline* and *ISI Web of Science* (*Science Citation Index Expanded*). The combination of the following keywords were used to search the studies: “*Staphylococcus aureus*”, “bacteremia”, “pneumonia”, “endocarditis”, “osteomyelitis”, “arthritis” “Quorum sensing”, “*agr* (accessory gene regulator)”, and “delta-hemolysin”. We excluded review articles, case reports and experimental studies. We also excluded colonization studies or epidemiological studies that did not report outcomes associated with *agr* functionality. Two authors (Shinwon Lee, Soon Ok Lee) independently performed the literature search and identified all studies potentially relevant for this review (Fig. 1).

### Data analysis

To analyse the association between the dysfunction of *agr* and outcomes of invasive *S. aureus* infections, we calculated odds ratios (ORs) comparing the odds of outcomes of *S. aureus* infection with dysfunctional *agr* with the odds of that with functional *agr*.

We used meta-analysis to obtain estimates of the odds of outcomes of invasive *S. aureus* infection with dysfunctional *agr* and presented ORs and their 95% confidence intervals (CIs) in random-effects model analysis. Meta-analysis results are presented as forest plots, and funnel plots were inspected to judge potential evidence for publication bias. We applied a two-sided significance level of 0.05. R version 3.3.2 (R Foundation for Statistical Computing, Vienna, Austria) and R package ‘meta’ were used for all statistical analyses^76,77^.

The number of patients and events for the dysfunctional versus functional *agr* group were extracted by one author (Shinwon Lee). The results were independently validated by another author (Soon Ok Lee). We extracted information on study designs, settings, patient characteristics, method for isolate collection (or selection) and the year of data collection to assess potential heterogeneity in the study populations. A formal risk assessment of the individual studies was judged according to the Newcastle–Ottawa Scale (NOS)^78^. To investigate whether dysfunctional *agr* is associated with poor outcome among cases of invasive disease, we compared outcomes, mainly 30-day mortality and the persistent bacteraemia. The included studies reported various end points as their outcomes, such as 30-day mortality, in-hospital mortality, *S. aureus* bacteraemia-attributable mortality, persistent bacteraemia, treatment failure, and development of complications (Table 1). We analysed data using three different outcomes, namely overall unfavourable outcomes, 30-day mortality and persistent bacteraemia. We defined overall unfavourable outcomes as comprehensive negative results from each study. Additionally, we performed post hoc analysis to determine the association between dysfunctional *agr* and mortality according to the site of infection. Information that was not mentioned in the original article was requested by contacting the authors directly. The study protocol is registered on PROSPERO with reference number ID: CRD42019134966.

## Supplementary information

Supplementary Information.
