# Supplementary material for: Dysfunctional accessory gene regulator (agr) as a prognostic factor in invasive Staphylococcus aureus infection: a systematic review and meta-analysis
Source: Sci Rep. 2020 Nov 26;10:20697. doi: 10.1038/s41598-020-77729-0 (PMC7691521; doi:10.1038/s41598-020-77729-0)

**Dysfunctional accessory gene regulator (*agr*) as a prognostic factor in invasive *Staphylococcus aureus* infection: A systematic review and meta-analysis**

Soon Ok Lee, Shinwon Lee, Jeong Eun Lee, Kyoung-Ho Song, Chang Kyung Kang, Yu Mi Wi, Rafael San-Juan, Luis E. López-Cortés, Alicia Lacoma, Cristina Prat, Hee-Chang Jang, Eu Suk Kim, Hong Bin Kim, Sun Hee Lee

**Supplemental table 1:** Newcastle-Ottawa Scale assessment of the quality of included studies – Cohort studies

| Study | Selection (0-4) | | | | Comparability (0-2) | Outcome (0-3) | | | Total (0-9) |
| --- | --- | --- | --- | --- | --- | --- | --- | --- | --- |
|  | Representativeness of the exposed cohort | Selection of the non- exposed cohort | Ascertainment of exposure | Demonstration that the outcome of interest was not present at the start of the study | Comparability of cohorts on the basis of the design or analysis | Assessment of outcome | Was follow-up long enough for outcomes to occur | Adequacy of the follow-up of cohorts |  |
| Fowler VG Jr. et al 2004 ^7^ |  | * | * | * | N/A | * | * | * | **6** |
| Moise PA et al 2007 ^36^ |  | * | * | * | N/A | * | * | * | **6** |
| McCalla C et al 2008 ^35^ | * | * | * | * | N/A | * | * | * | **7** |
| Walraven CJ et al 2011 ^39^ | * | * | * | * | N/A | * | * | * | **7** |
| Schweizer ML et al 2011 ^29^ | * | * | * | * | N/A | * | * | * | **7** |
| Sharma-Kuinkel BK et al 2012 ^38^ | * | * | * | * | N/A | * | * | * | **7** |
| Park SY et al 2013 ^30^ |  | * | * | * | N/A | * | * | * | **6** |
| Chong YP et al 2013 ^50^ | * | * | * | * | N/A | * | * | * | **7** |
| Jang HC et al 2013 ^24^ | * | * | * | * | N/A | * | * | * | **7** |
| Casapao AM et al 2013 ^34^ |  | * | * | * | N/A | * | * | * | **6** |
| Wi YM et al  2015 ^27^ | * | * | * | * | N/A | * | * | * | **7** |
| Hu HC et al 2015 ^41^ |  | * | * | * | N/A | * | * | * | **6** |
| Kang CK et al 2015 ^31^ | * | * | * | * | N/A | * | * | * | **7** |
| López-Cortés LE et al 2015 ^73^ | * | * | * | * | N/A | * | * | * | **7** |
| McDanel JS et al 2015 ^77^ | * | * | * | * | N/A | * | * | * | **7** |
| Kang CK et al  2017 ^32^ |  | * | * | * | N/A | * | * | * | **6** |
| Gomes-Fernandes M 2017 ^33^ | * | * | * | * | N/A | * | * | * | **7** |
| Sullivan SB et al  2017 ^55^ | * | * | * | * | N/A | * | * | * | **7** |
| San-Juan R et al 2017 ^37^ |  | * | * | * | N/A | * | * | * | **6** |
| Yang CC et al 2018 ^40^ |  | * | * | * | N/A | * | * | * | **6** |
| Fernández-Hidalgo N et al 2018 ^42^ |  | * | * | * | N/A | * | * | * | **6** |

**Abbreviation**

*Selection*

1) Representativeness of the exposed cohort: a) truly representative of the average patient with invasive *S. aureus* infection*****, b) somewhat representative of the average patient with invasive *S. aureus* infection*****, c) selected group of patients with SAB only, d) no description of the derivation of the cohort

2) Selection of the non-exposed cohort: a) drawn from the same population as the exposed cohort *****, b) drawn from a different source, c) no description of the derivation of the non-exposed cohort

3) Ascertainment of exposure: a) secure record, b) structured interview *****, c) written self-report, d) no description

4) Demonstration that the outcome of interest was not present at the start of the study: a) yes ***,** b) no

*Comparability*

1) Comparability of cohorts on the basis of the design or analysis; a) study controls for ----------*** (select the most important factor)**, b) study controls for any additional factor **** (This criterion could be modified to indicate specific control for a second important factor)**

*Outcome*

1) Assessment of outcome, a) independent or blind assessment *****, b) record linkage *****, c) self-report , d) no description

2) Was follow-up long enough for outcomes to occur, a) yes *****, b) no

3) Adequacy of the follow-up of cohorts, a) complete follow-up - all subjects accounted for *****, b) subjects lost to follow-up unlikely to introduce bias - small number lost > 20%, c) follow-up rate < 20% and no description of those lost, d) no statement

**Supplemental table 2:** Frequency of sites of infections of invasive *S. aureus* infections among included studies. This analysis was conducted before subgroup analysis by sites of infections to identify most common sites of infection.

| **study** | **Site of infection (Numbers)** |
| --- | --- |
| Jang HC 2013 (N=307) | Skin and soft tissue (65)  Bone and joint (26)  Catheter-related bloodstream (101)  Lower respiratory tract (29)  Intra-abdominal (23)  Unknown 65 |
| Wi YM 2015 (N=146) | Skin and soft tissue (29)  Bone and joint (10)  Catheter-related bloodstream (22)  Lower respiratory tract (18)  Intra-abdominal (11)  Urinary tract (14)  Endocarditis (19)  CNS infection (6)  Unknown (22) |
| Kang CK 2015 (N=171) | Skin and soft tissue (26)  Bone and joint (11)  Catheter-related bloodstream (49)  Lower respiratory tract (21)  Intra-abdominal (7)  Surgical wound (27)  Unknown (20) |
| Lopez-Cortez LE 2015 (N=135) | Skin and soft tissue (18)  Catheter-related bloodstream (61)  Lower respiratory tract (10)  Endocarditis (11)  Unknown (24) |
| Gomes-Fernandes M 2017 (N=18) | Lower respiratory tract (18) |
| Sullivan SB 2017 (N=252) | Skin and soft tissue (34)  Catheter-related bloodstream (53)  Lower respiratory tract (40)  Other (72)  Unknown (53) |
| Hu HC 2015 (N=48) | Catheter-related bloodstream (7)  Lower respiratory tract (24)  Surgical wound (3)  Other (3)  Unknown (11) |
| McDanel JS 2015 (N=75) | Lower respiratory tract (75) |
| Kang CK 2017 (N=152) | Skin and soft tissue (30)  Bone and joint (29)  Catheter-related bloodstream (35)  Lower respiratory tract (10)  Endocarditis (11)  Unknown (14) |
| Yang CC 2018 (N=147) | Skin and soft tissue (24)  Bone and joint (19)  Catheter-related bloodstream (56)  Lower respiratory tract (34)  Intra-abdominal infection (1)  Urinary tract infections (3)  Cardiovascular, mainly endocarditis (8)  Unknown (23) |
| Fernandez-Hidalgo N 2018 (N=213) | Endocarditis (213) |
| **Total** | **Skin and soft tissue (226)**  **Bone and joint (95)**  **Catheter-related bloodstream (384)**  **Lower respiratory tract (279)**  **Infective endocarditis (264)**  Intra-abdominal infection (51)  Surgical wound (30)  Urinary tract (17)  CNS infection (6)  Unknown (232) |

| **Supplementary Figure 1:** The results for the association of *agr* dysfunction with overall mortality in patients with invasive *S. aureus* infection: (A) total, (B) MRSA, and (C) MSSA  **(A)**  **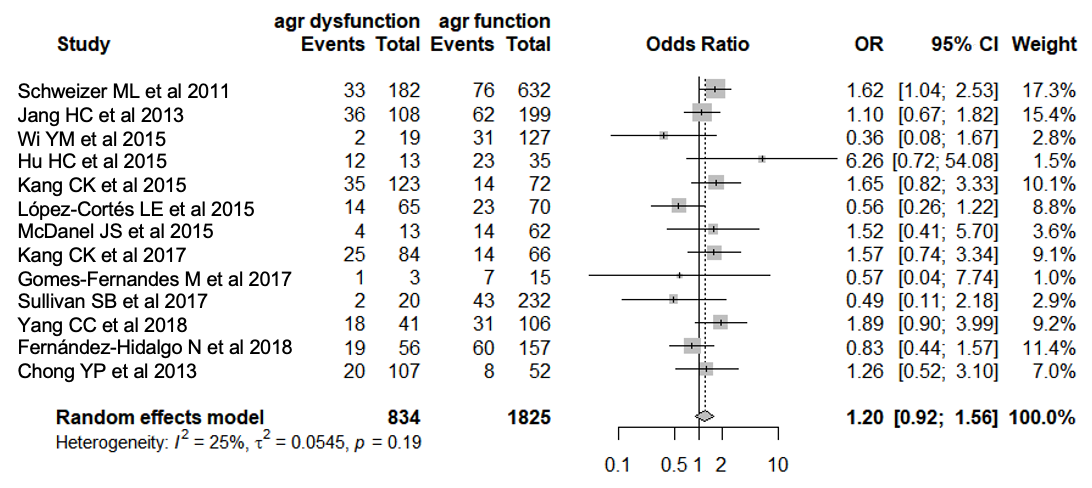**  **P=0.182**  **(B)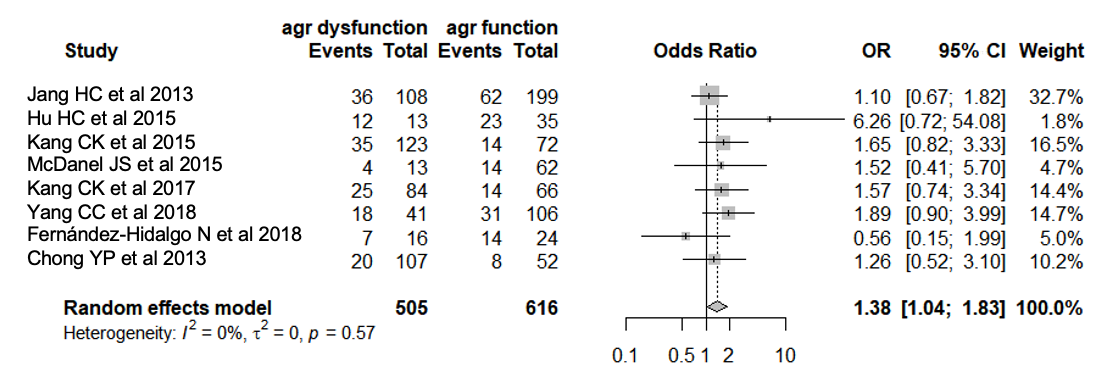**  **P=0.028**  **(C)**  **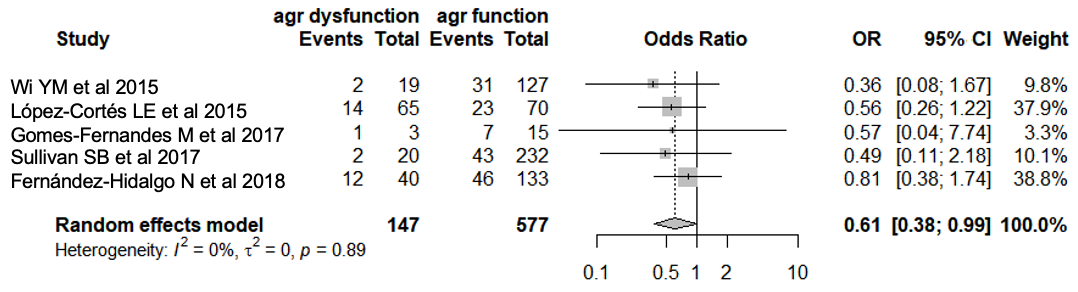**  **P=0.043**  **Supplementary figure 2.** The result for association with *agr* dysfunction and mortality in patients with invasive MSSA infection by sites of infections.   \| **(A) Central-line-associated bloodstream infection**  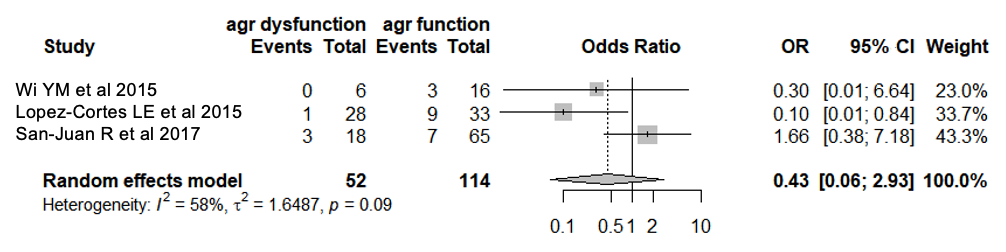 \| \| --- \| \| **P=0.390** \| \| **(B) Infective endocarditis**  **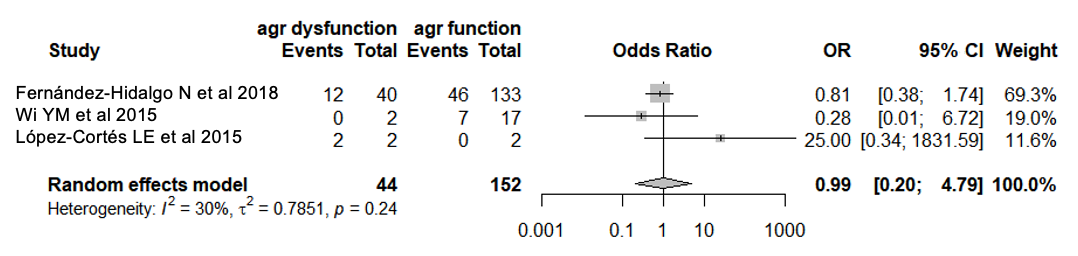** \| \| **P=0.987** \| \| **(C) Lower respiratory infection**  **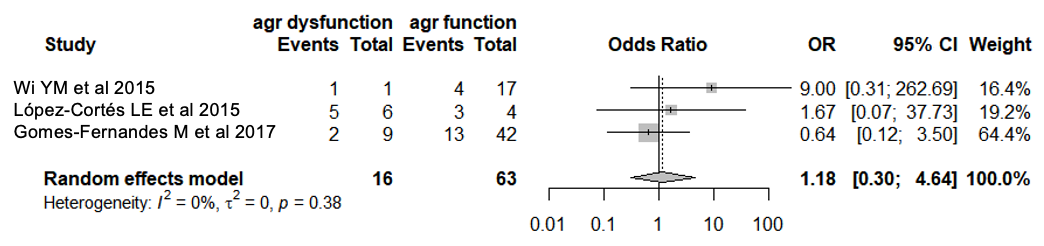** \| \| **P=0.809** \| \| **(D) Skin and soft tissue infection**  **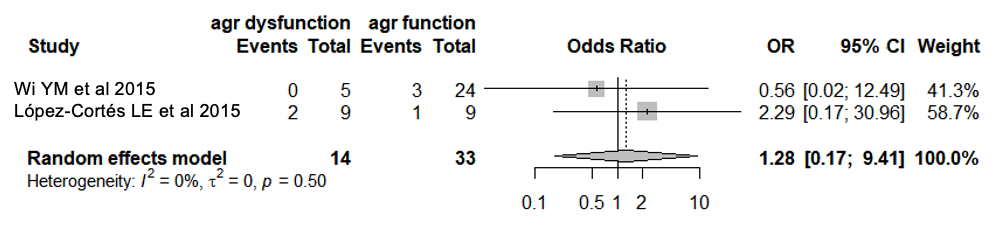** \| \| **P=0.810** \| \| **(E) Bone and joint infection**  **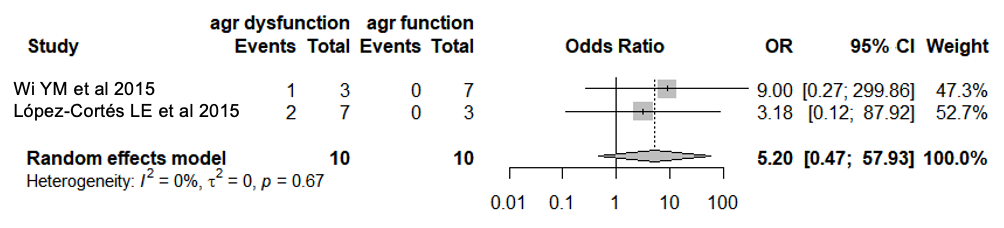** \| \| **P=0.180** \|   **Supplementary Figure 3.** The results for the association of *agr* dysfunction with overall outcome in patients with *S. aureus* bacteraemia: (A) total, (B) MRSA, and (C) MSSA  **(A)**  **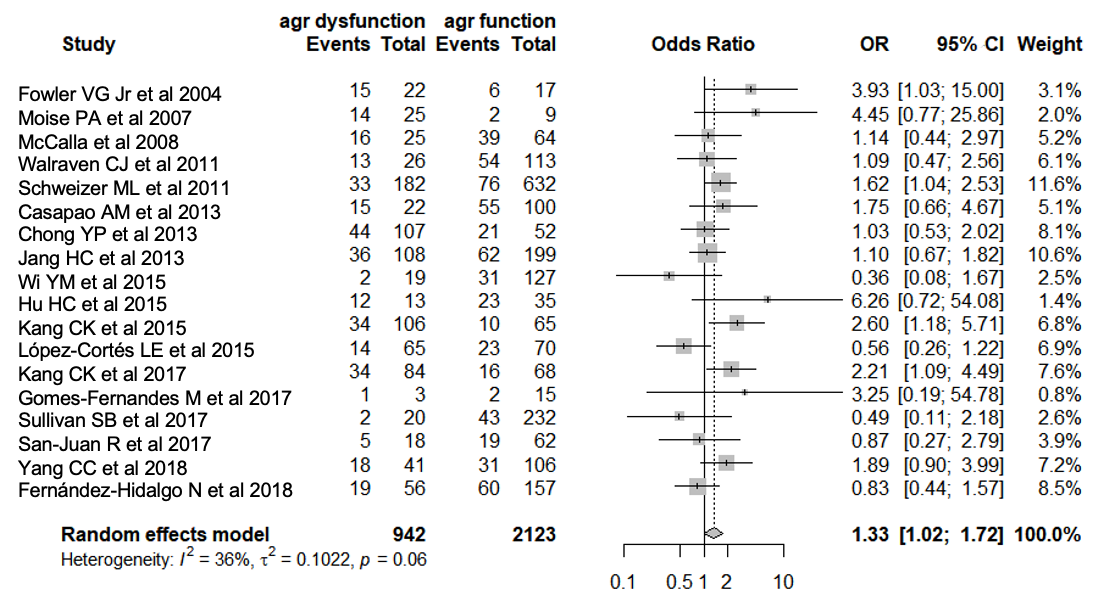** |
| --- | --- | --- | --- | --- | --- | --- | --- | --- | --- | --- |
| **P=0.035** |
| **(B)**  **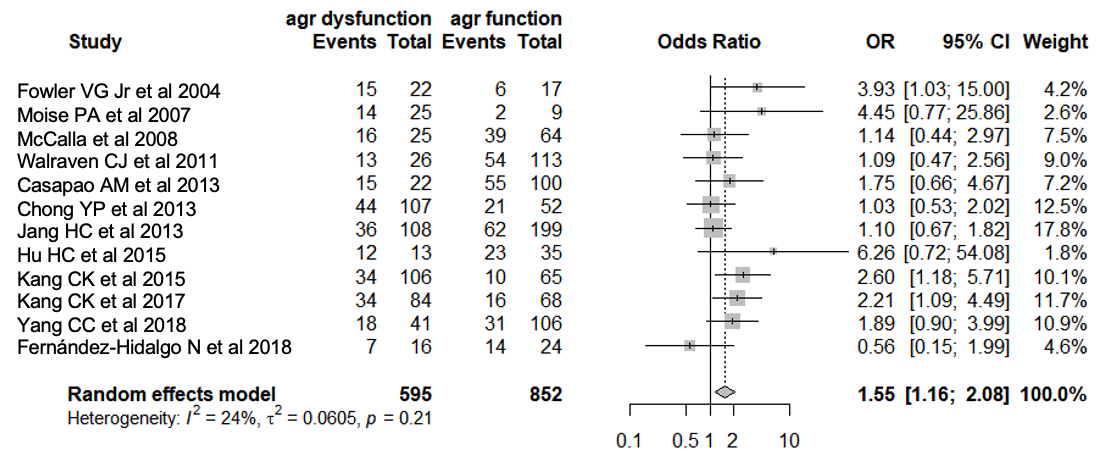** |
| **P=0.032** |
| **(C)**  **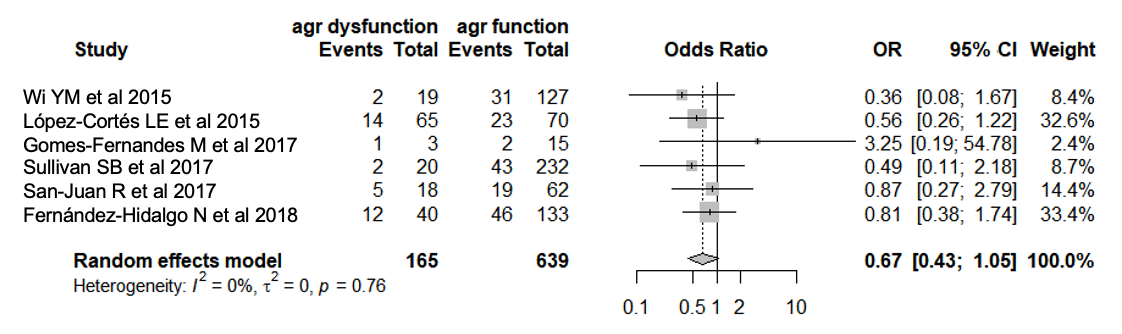**  **P=0.078** |

**Supplementary Figure 4:** Assessment of publication bias for the association of agr dysfunction with overall outcome in patients with invasive *S. aureus* infection: (A) total, (B) MRSA, and (C) MSSA.


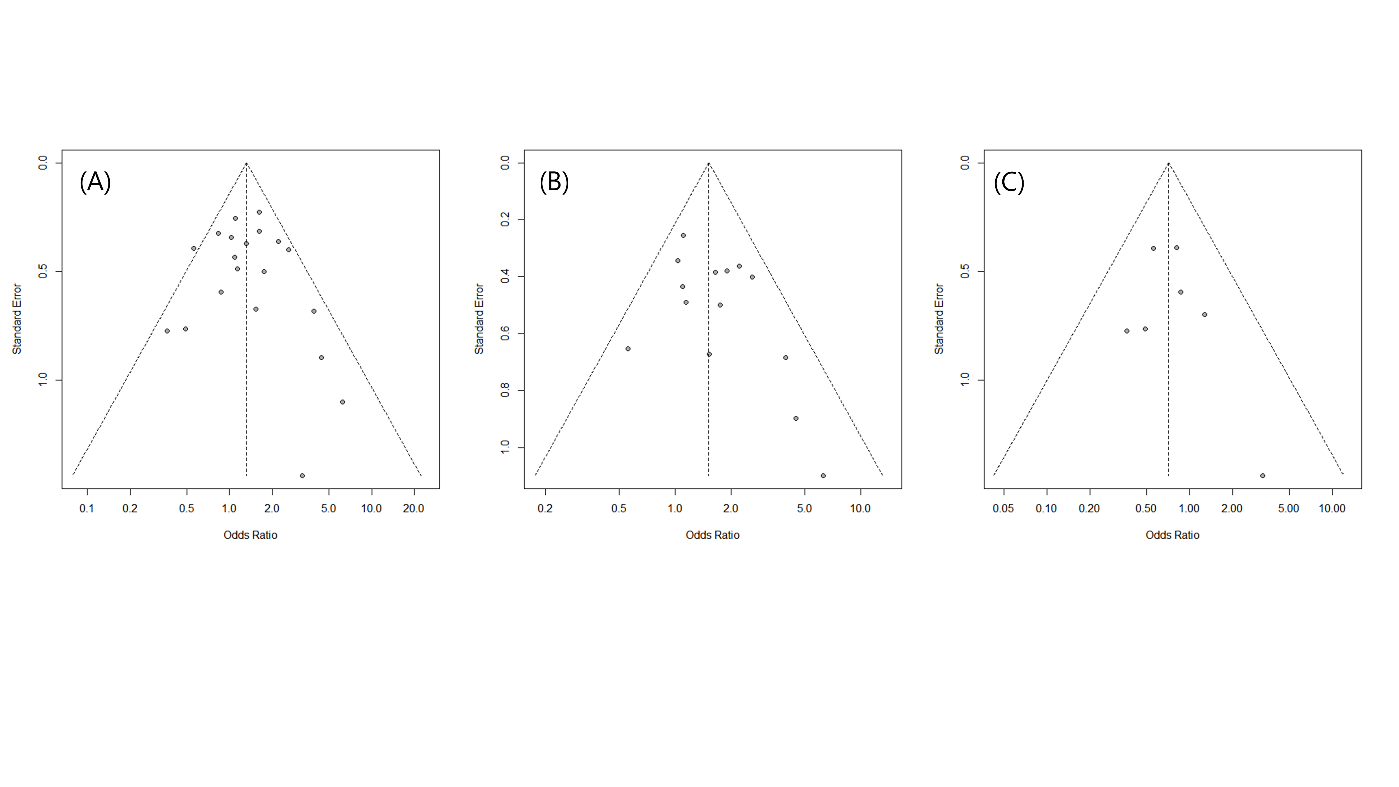


**Supplementary Figure 5:** Assessment of publication bias for the association of agr dysfunction with 30-day mortality in patients with invasive S. aureus infection: (A) total, (B) MRSA, and (C) MSSA.


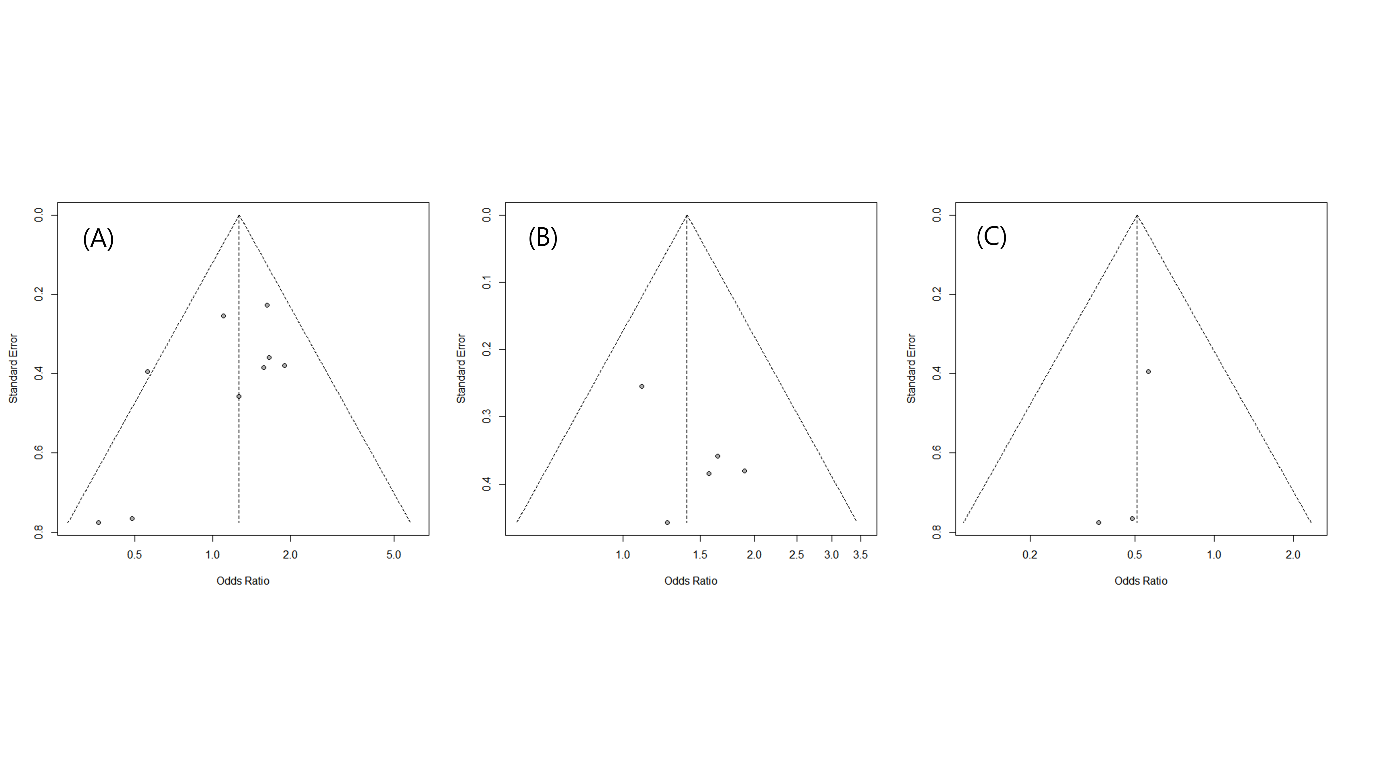


**Supplementary Figure 6:** Assessment of publication bias for the association of agr dysfunction with mortality in patients with invasive MRSA infection according to the site of infection: (A) lower respiratory tract infection, (B) bone and joint infection, (C) central-line-associated bloodstream infection, (D) skin and soft tissue infection, and (E) infective endocarditis.


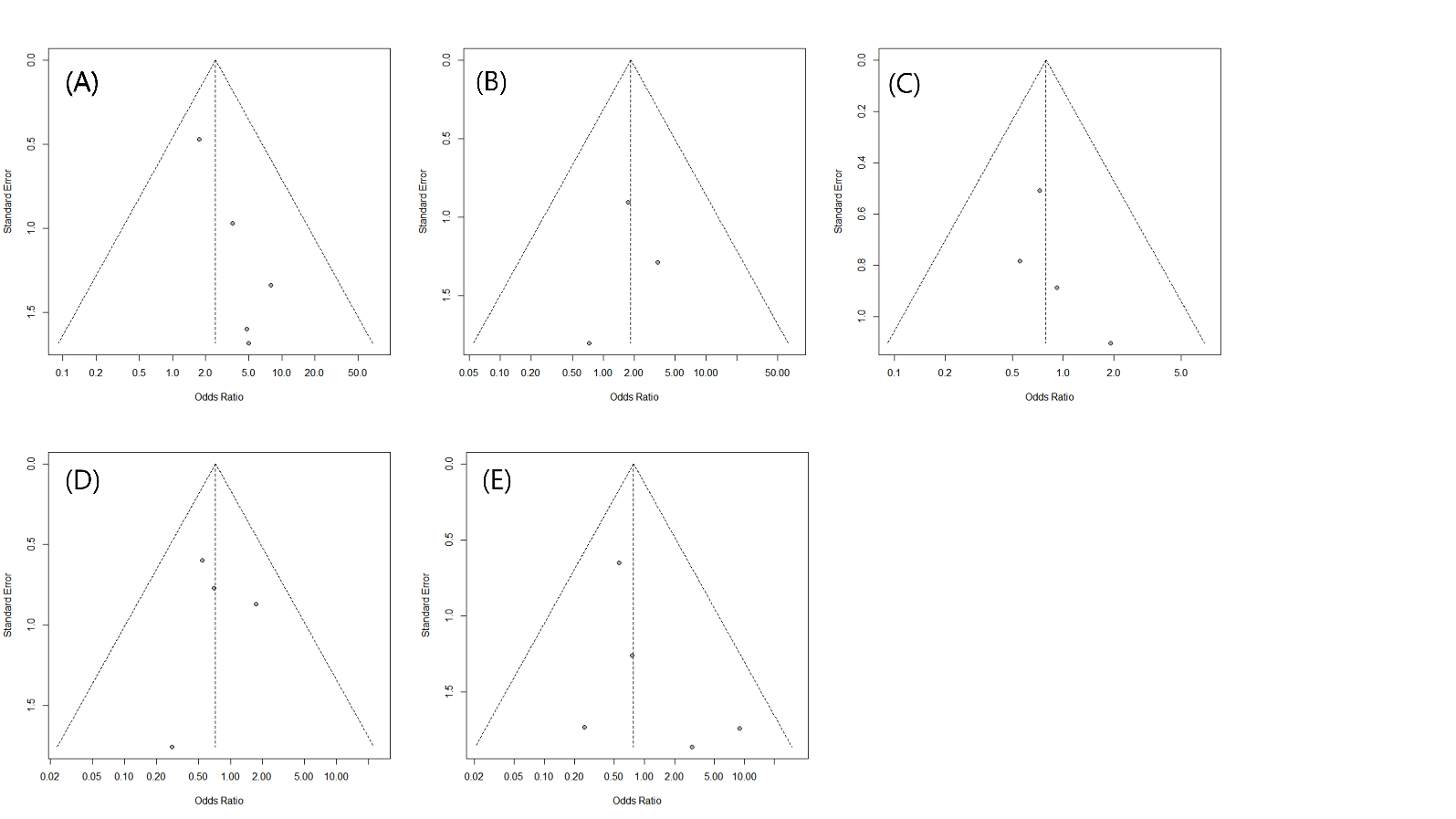


**Supplementary Figure 7:** Assessment of publication bias for the association of agr dysfunction with persistent bacteraemia in patients with *S. aureus* bacteraemia: (A) total, (B) MRSA, and (C) MSSA.


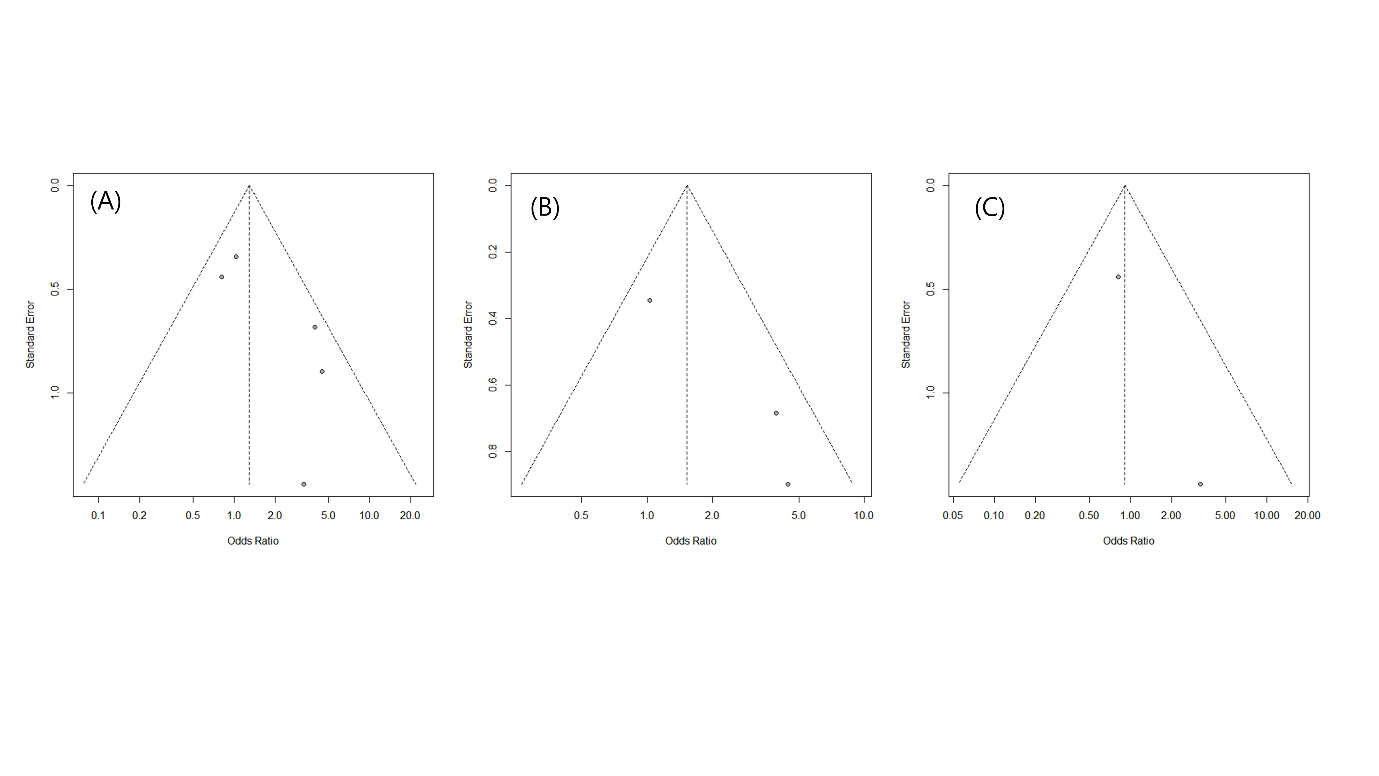

Supplement: Supplementary file 1 — Supplementary Information. [file 41598_2020_77729_MOESM1_ESM.docx]
